# Regression—A Means, Not an End

**DOI:** 10.1002/sim.70000

**Published:** 2025-02-04

**Authors:** Robert W. Platt

**Affiliations:** ^1^ Department of Epidemiology, Biostatistics, and Occupational Health McGill University Montreal Quebec Canada

I congratulate Carlin and Moreno‐Betancur (C&M) on this important article [1]. They have thoughtfully described use and misuse of regression models and make concrete suggestions for improvement of teaching of statistical methods. In this commentary, I will amplify a few points from this important piece and raise two related complementary issues.

C&M review important basic concepts of regression and then focus their attention on the need for clear objectives when using regression methods, and on how teaching of regression is often unclear (at best) with respect to those goals. In line with Hernán and colleagues, they focus on three specific objectives of statistical analysis: description, prediction, and causation [2], and describe several settings in which regression is used but where the goals of the analysis are non‐specific. Such lack of specificity of goals affects methods across applied statistics and data science, not just regression, but given its ubiquity regression is certainly one of the principal problem areas. In my experience, it is far too often that researchers start on a regression exercise without clear goals, leading to incoherent interpretation of regression coefficients as measures of causal effects, or worse. As C&M note, this is inappropriate for a number of reasons, including issues like the “table 2 fallacy” [3]. There are plenty of examples of these errors; I thank the authors for citing some of their own, and I suspect a review of my own work would find similar problems!

Another limitation of how we use regression has to do with the parameters themselves, and our interpretation of them, even when a regression model may be appropriate. For example, the development of logistic regression was a breakthrough in the analysis of case–control studies [4, 5]. However, this led to the odds ratio, estimated by the exponentiated (logistic) regression coefficient, becoming the default parameter to represent associations between an exposure and a binary outcome regardless of the study design. In my view, this occurred because of the relative computational simplicity of logistic regression when it was developed, rather than any particular relevance of the parameter itself; certainly, for cohort and other designs there are better options. This misuse is still prevalent, despite the obvious limitations of the odds ratio in this regard and improvements in estimation of marginal relative risks and risk differences [6]. Recent developments related to estimands [7] will undoubtedly accelerate the discussion of appropriate parameters; the estimand represents a subject‐relevant target parameter, often not corresponding to a regression coefficient, that directly addresses a research question. This should push researchers to consider appropriate methods to estimate a relevant target parameter, rather than defaulting to the readily available parameters of the regression model.

I strongly agree with the authors that regression is best used as a tool to achieve a goal, rather than as a result or goal itself; if one has a very clear understanding of the objective of a study, then regression can be appropriately used and can inform a problem. I appreciate C&M's thoughts on roadmaps for studies that depend on the objective of the study. These frameworks clarify the role of regression and modeling more broadly as a tool that plays a part in the process, rather than an end. The Causal Roadmap [8] is a framework developed in concert with targeted learning [9] providing a similar approach to causal inference; essentially, it provides a set of steps, focused on a causal question, that includes analyses appropriate for this target parameter. Targeted learning and related semiparametric methods allow focus on the estimand, and use statistical or machine learning models to fit nuisance models, components of which are used to build an estimator for the estimand of interest.

Finally, I would point out that one consequence of the focus on regression is that too much of modern teaching of statistics has been built around analysis first, ignoring the role of study design and sampling. I think too much of our teaching underplays the central role of study design in statistics. Regression and other analytic tools must be taught hand‐in‐hand with study design, as the study design has a very important effect on the identifiability and validity of parameters, and on interpretation. Robins' classic paper “Data, Design, and Causal Inference” describes a theoretical setting in which analyses of the same variables leads to three different conclusions depending on the underlying design [10], showing the importance of design and sampling to any interpretation. A critical part of any statistical roadmap must be respect for the design, including sampling and external sources of structure in the data. In descriptive and predictive settings, this is important; the sample must be representative of the target population that the researcher wants to describe or make predictions on. For causal goals, design is critically important, as poor design choices can lead to inappropriate and biased analyses. The target trial framework popularized by Hernan and colleagues for identification and estimation of causal effects from observational studies [8, 11] focuses the researcher's attention on the design choices needed to minimize biases such as immortal time bias [12].

In conclusion, C&M have made an important contribution to the teaching of statistics, and I hope that this piece leads to renewed discussions of best practices in teaching. I agree with the authors that reform of teaching and use of regression methods is necessary, and look forward to continuing this discussion. Ideally, I hope that we can move to a framework where the question and the design drive the analysis, and not the other way around.

## Conflicts of Interest

The author declares no conflicts of interest.

## Data Availability

Data sharing not applicable to this article as no datasets were generated or analysed during the current study.
